# Supplementary material for: Association between oral microbiome and seven types of cancers in East Asian population: a two-sample Mendelian randomization analysis
Source: Front Mol Biosci. 2023 Nov 21;10:1327893. doi: 10.3389/fmolb.2023.1327893 (PMC10702768; doi:10.3389/fmolb.2023.1327893)
Supplement: Supplementary file 1 [file DataSheet1.ZIP › Supplementary Materials/MR plots of saliva/saliva═╝/Prostate cancer/pheno.2386_to_prostate cancer_forest.pdf]

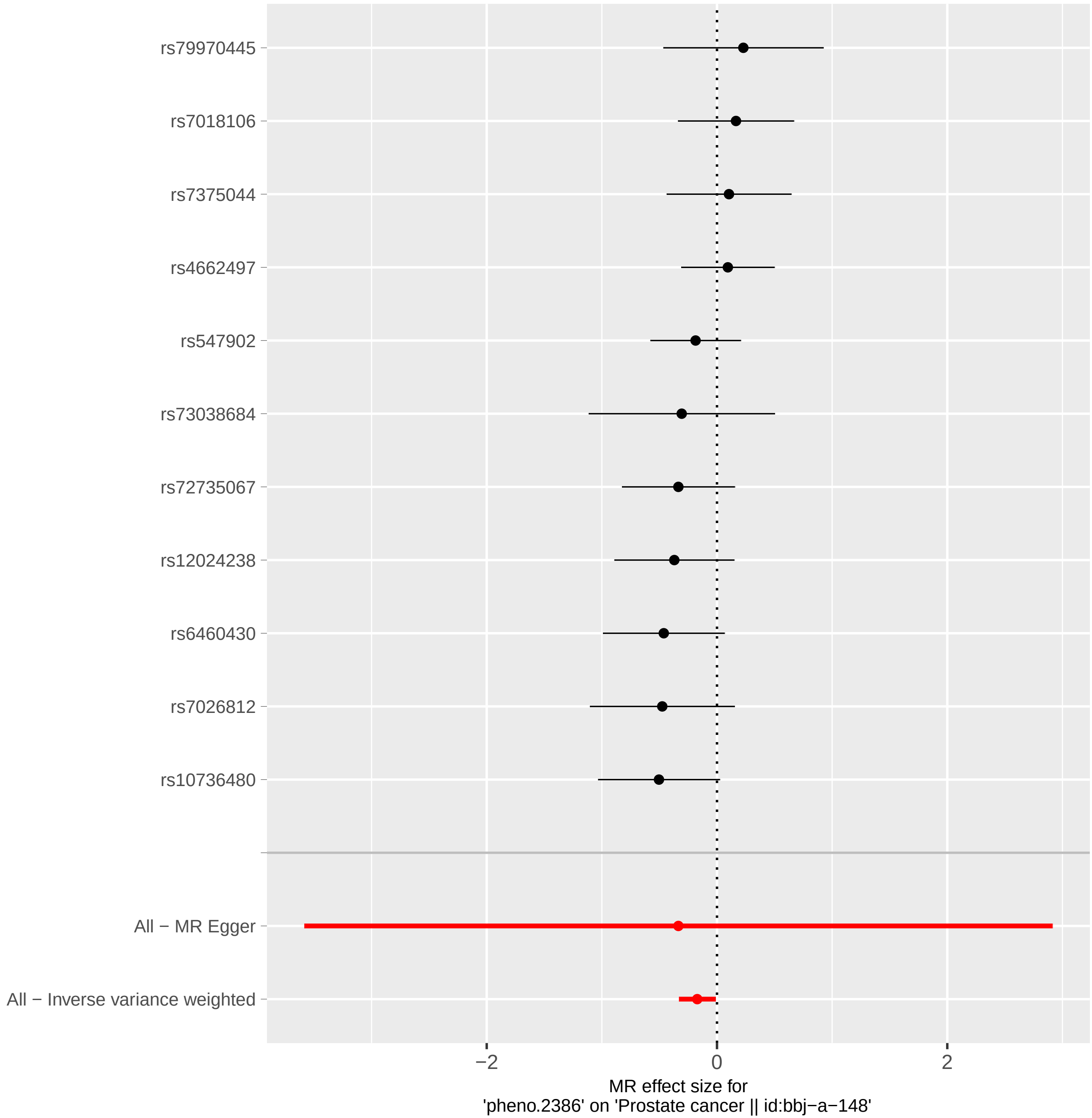

rs79970445
rs7018106
rs7375044
rs4662497
rs547902
rs73038684
rs72735067
rs12024238
rs6460430
rs7026812
rs10736480
All − MR Egger
All − Inverse variance weighted
−2
0
2
MR effect size for
'pheno.2386' on 'Prostate cancer || id:bbj−a−148'
